# Critical roles of TRPV2 channels, histamine H1 and adenosine A1 receptors in the initiation of acupoint signals for acupuncture analgesia

**DOI:** 10.1038/s41598-018-24654-y

**Published:** 2018-04-25

**Authors:** Meng Huang, Xuezhi Wang, Beibei Xing, Hongwei Yang, Zheyan Sa, Di Zhang, Wei Yao, Na Yin, Ying Xia, Guanghong Ding

**Affiliations:** 1 Shanghai Key Laboratory of Acupuncture Mechanism and Acupoint Function, Fudan University, Shanghai, 200433 China; 2 Department of Aeronautics and Astronautics, Fudan University, Shanghai, 200433 China; 3 Shanghai Research Center for Acupuncture and Meridian, Shanghai, 201203 China; 4 Fujian Academy of TCM, Fuzhou, Fujian, 350003 China

## Abstract

Acupuncture is one of the most promising modalities in complimentary medicine. However, the underlying mechanisms are not well understood yet. We found that in TRPV2 knockout male mice, acupuncture-induced analgesia was suppressed with a decreased activation of mast cells in the acupoints stimulated. The mast cell stabilizer sodium cromolyn could suppress the release of adenosine in the acupoints on male rats. A direct injection of adenosine A1 receptor agonist or histamine H1 receptor agonist increased β-endorphin in the cerebral-spinal fluid in the acute adjuvant arthritis male rats and thus replicated the analgesic effect of acupuncture. These observations suggest that the mast cell is the central structure of acupoints and is activated by acupuncture through TRPV2 channels. The mast cell transduces the mechanical stimuli to acupuncture signal by activating either H1 or A1 receptors, therefore triggering the acupuncture effect in the subject. These findings might open new frontiers for acupuncture research.

## Introduction

Acupuncture is a traditional Chinese medical therapy that has a long history and remains widely used in contemporary clinical practice. Modern medical research has demonstrated that acupuncture has a significant therapeutic effect on chronic pain^1^, musculoskeletal pain^2^, knee osteoarthritis^3,4^, depression^5,6^, rhinitis^7^, postoperative rehabilitation of rectal cancer^8^, stroke^9,10^, hypertension^11^, angina pectoris^12^, and constipation^13^. In 2002, the WHO noted that acupuncture had an efficacy superior to that of control groups for up to 63 diseases, with significant efficacy for 28 of them^14^.

At present, the commonly accepted consensus is that acupuncture triggers systemic responses, including responses in the nervous system, by physically stimulating specific sites (called acupuncture points or acupoints) on the surface of the human body, thereby regulating human body functions to eventually achieve a therapeutic effect. For example, stimulating the extremities can trigger the regulatory action of the cardiovascular system^15^, and stimulating local acupoints can induce a systemic analgesic effect^16^. However, we still do not know much about acupuncture-triggered local acupoint response mechanisms. Studying the effects of changes to an acupoint’s local tissue environment after acupuncture on the generation of acupuncture-initiated signals may reveal the mystery of acupoint signals.

Acupuncture is a mechanical force stimulation. Deformation of tissue leads to local damage as well as mechanical signal transduction. By way of dissection and ultrasonic image observation^17–20^, Langevin *et al*. found that local muscles at acupoints were intertwined with the connective tissues. They suggested that such a deformation of local tissue was a hallmark of “acquiring qi” through acupuncture and was also the initial factor in the initiation of an acupuncture signal. Yu *et al*. destroyed the collagen structure at acupoints by injecting collagenase into the acupoints of an animal model^21^. They found that the acupuncture analgesic effect generated by the acupoint tissue after such a treatment decreased dramatically when the acupuncture was performed again, suggesting that the collagen fibres in the acupoints play an important role in mediating and transmitting acupuncture mechanical force. However, the target of the mechanical force mediated by collagen is still unclear. In a previous *in vitro* experiment, we found that mast cells could be activated by mechanical stimulation through the TRPV2 protein on their membrane^22^. In the present study, we used TRPV2 gene knockout mice and studied the activation process of local key cells at the acupoints implicated in TRPV2 protein-involved acupuncture effects.

Regarding the tissue environmental changes in acupoints, Goldman *et al*. found that acupuncture could result in an abnormal increase of adenosine concentration in acupoint tissue, which could activate local adenosine receptor A1 and produce a corresponding acupuncture analgesic effect^23^. If adenosine receptor A1 were knocked out, the acupuncture analgesic effect would be significantly reduced or would disappear. This finding suggests that changes in adenosine concentration at acupoints following acupuncture meaningfully influence the subsequent physiological effect. However, Goldman *et al*. could not explain the specific pathway by which acupuncture-stimulated adenosine concentrations increased at the acupoints. In the present study, we investigated the relationship between mast cells and local adenosine signalling at acupoints. We used the mast cell degranulation inhibitor sodium cromolyn to study whether the degranulation of mast cells affects the acupuncture-induced increase of adenosine concentration at acupoints. In addition, we used an adenosine receptor A1 agonist to study whether the adenosine signal triggers acupuncture’s effect by activating mast cells.

Zhang *et al*. found that acupuncture caused an aggregation and degranulation of mast cells at acupoints, which was correlated with the acupuncture analgesic effect^24^. The granules from degranulated mast cells contain large amounts of bioactive substances, such as histamine, substance P, 5-HT, IL2, and cell chemokines^25^, through which histamine can trigger a series of physiological responses via the histamine H1 receptor in local tissue^26^. In previous studies, we found that the injection of histamine at acupoints can induce the acupuncture analgesic effect^27^, and we used specific antagonists and inhibitors of the histamine H1 receptor to study the interacting receptor of histamine released from the granules of degranulated mast cells.

A central mechanism for the acupuncture analgesic effect has been widely accepted and is generally believed to be associated with the release of opioid peptides in the brain^28^. Studies have shown that acupuncture can lead to an increase in the concentration of β-endorphin (EDP) in the cerebrospinal fluid of patients with pain^29^. We chose EDP in cerebrospinal fluid to study the relationship between the local initiation mechanism at acupoints triggered by acupuncture and the release of central opioid peptides.

Comparison of functional difference between acupoints and non-acupoint had been subject of another research^30^. The present work investigates how acupuncture stimulation is converted into effective acupuncture signals at acupoint, establishes the signal initiation pathway of tissue collagen-TRPV protein-mast cells, and clarifies the important role played by adenosine-histamine at acupoints in the initiation of acupoint signals.

## Results

### Effects of the TRPV2 channel on the activation of mast cells at acupoints and on the acupuncture analgesic effect

**Figure 1:**
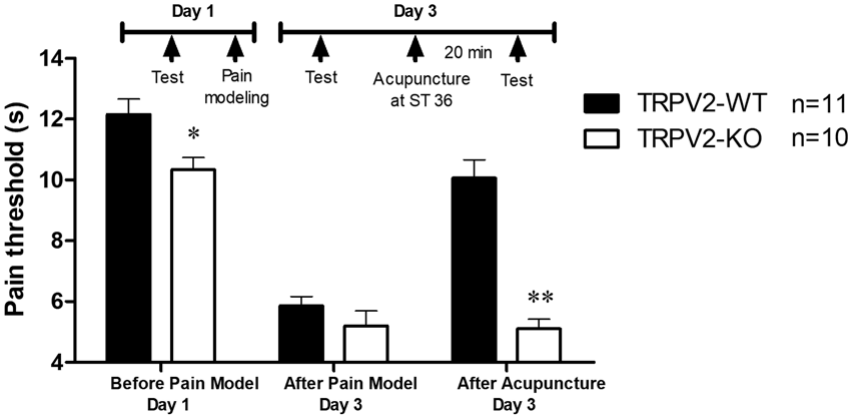
Different acupuncture effects between TRPV2 knockout (KO) and wild-type (WT) mice. The pain threshold data are presented as the mean ± s.e.m. On day 1, the inflammatory pain model was established. Before establishing the model, the pre-modelling pain threshold was measured. On day 3, the first, post-modelling pain threshold was measured. Acupuncture treatment was performed on the left Zusanli acupoint (ST36) for 30 min, and 20 min later, the post-acupuncture pain threshold was measured. TRPV2-KO is the gene knockout group; TRPV2-WT is the littermate wild-type group. The knockout of the TRPV2 gene inhibited the analgesic effect triggered by acupuncture in this inflammatory-pain mouse model. *vs TRPV2-WT P < 0.05, **vs TRPV2-WT P < 0.01.

As immune cells, mast cells can be activated by various stimuli, including physical factors, chemical factors and immunoglobulin. We demonstrated the activation of mast cells by mechanical stimulation in *in vitro* experiments and found that the mechanosensitive channel protein TRPV2 expressed on the membrane of mast cells is primarily involved in the activation process^22^. On the basis of this finding, we used transgenic mice to compare the difference in the acupuncture analgesic effect in wild-type-TRPV2 (TRPV2-WT) mice and TRPV2-knockout (TRPV2-KO) mice with inflammatory pain. After generating the model, the pain thresholds of the animals in the two groups did not show a significant difference. After acupuncture treatment, the pain threshold of the KO group was significantly lower than that of the WT group (P < 0.01, vs TRPV2-WT, see Fig. 1), with the acupuncture analgesic effect in the TRPV2-KO mice having been inhibited significantly.

**Figure 2:**
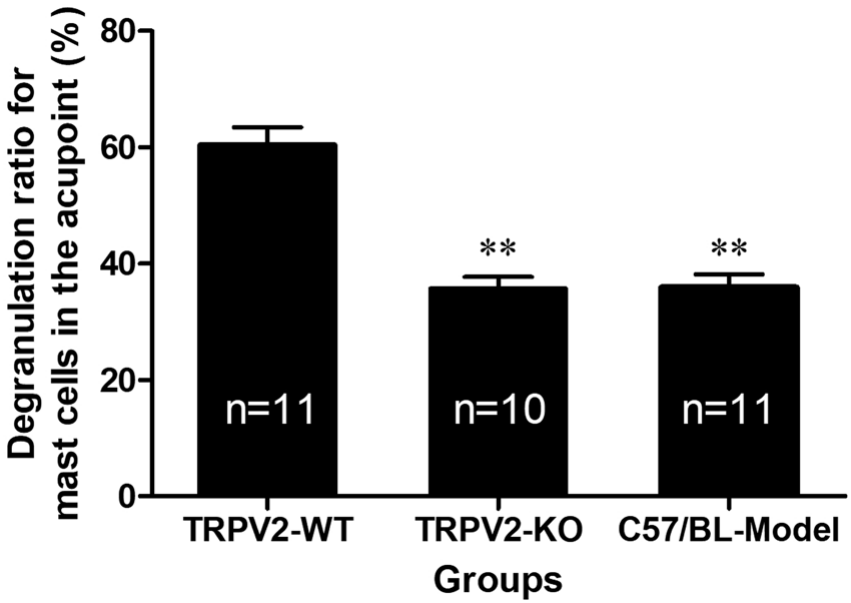
Difference in degranulation ratios of mast cells at ST36 of TRPV2-knockout and wild-type mice after acupuncture. The data for degranulation of mast cells are presented as the mean ± s.e.m. The TRPV2-KO group is the gene knockout group; the TRPV2-WT group is the littermate control group. **vs. TRPV2-WT P < 0.01.

To study the effect of TRPV2 knockout on the activation of local mast cells at acupoints, we examined paraffin sections of the ST36 local skin of mice in the two groups. Toluidine blue staining was used to label the mast cells and obtain the ratio of the degranulated mast cells versus the total number of mast cells by counting under the microscope (Fig. 2). The TRPV2-KO group is the gene knockout mouse group, in which the degranulation of mast cells after acupuncture was significantly lower than that of wild-type mice (P < 0.01, vs TRPV2-WT, see Fig. 2). Acupuncture induced mast cell degranulation in TRPV2-WT group (P < 0.01, vs. C57/BL-Model). As shown in Fig. 2, the acupuncture-induced degranulation of mast cells was significantly lower in the TRPV2-KO group than that of wild-type mice (P < 0.01, vs. TRPV2-WT; P > 0.05, vs. C57/BL-Model; Fig. 2). This result supported our conclusion from the *in vitro* experiment, suggesting that the mechanosensitive protein TRPV2 is indeed involved in the mast cell activation process triggered by acupuncture at acupoints.

### Effects of mast cell activation on the increase in adenosine concentrations at acupoints and on the acupuncture analgesic effect

Goldman *et al*. reported that acupuncture led to increases in adenosine concentrations at acupoints and suggested that such increases were the basis for the acupuncture analgesic effect^23^. However, their study did not explain the source of adenosine molecules and the specific pathway implicated in the increases in adenosine concentrations at the acupoints that was activated by acupuncture. Our studies found that the increase in adenosine was influenced by mast cells.

**Figure 3:**
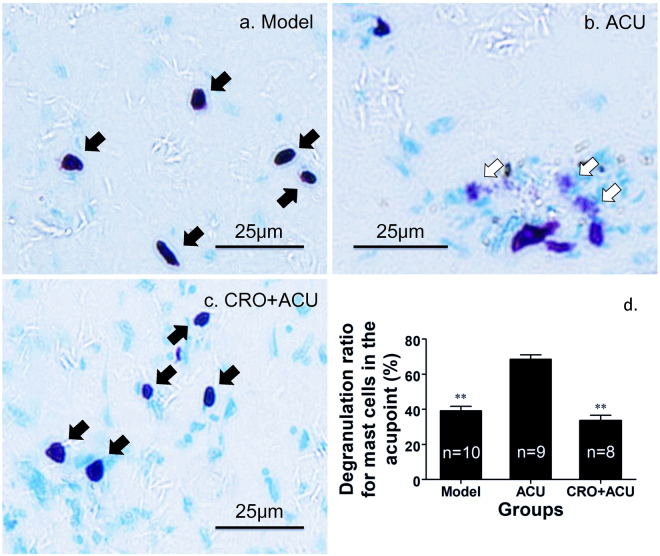
The degranulation of mast cells at ST36 induced by acupuncture and its inhibition by sodium cromolyn. After each experimental rat was sacrificed, a 5 × 5 × 5 mm^3^ volume of skin and muscle tissue from the ST36 acupoint was taken. After 48 hours of fixation in formalin solution, the tissue was cut into 5 μm paraffin sections, with the section plane perpendicular to the skin’s surface. After staining with toluidine blue, the mast cells were dark purple in colour as shown in (**a**) for Model group, (**b**) for ACU group and (**c**) for CRO + ACU group.The acupuncture time at the ST36 acupoints of the animals in the ACU and CRO + ACU groups was 30 min. Sodium cromolyn solution was injected at the acupoint 5 min before acupuncture for the CRO + ACU group. After acupuncture, degranulation of the mast cells was detected in the tissue at the acupoint, as shown by the hollow arrows in the figure. The cell boundaries of mast cells were blurred, and scattered granules were visible in the surrounding regions. In the specimens in the Model group and CRO + ACU group, the mast cells were found to manifest generally clear boundaries, as shown by the black arrows in the figure. (**d**) The difference of degranulation ratios amomg these three groups is shown in bar graph. The data are presented as the mean ± s.e.m. **vs. ACU P < 0.01. Sodium cromolyn was found to inhibit the mast cell degranulation triggered by acupuncture.

**Figure 4:**
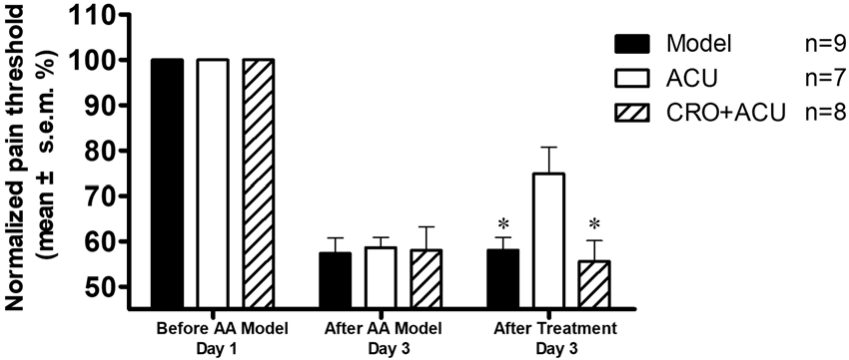
Effect of sodium cromolyn injection on acupuncture analgesia. Pain threshold was normalized according to pain thresholds determined prior to establishing the AA model; the data are presented as the mean ± s.e.m. in the figure. On day 1, the AA model was established. Before establishing the model, the pre-modelling pain threshold was measured. On day 3, first, the post-modelling pain threshold was measured, and the post-treatment pain threshold was measured 20 min after treatment. For the ACU group, acupuncture was performed at the ST36 acupoint for 20 min. For the CRO + ACU group, sodium cromolyn solution was injected locally at the acupoint 5 min before acupuncture. The Model group was restrained for 20 min. Sodium cromolyn was found to inhibit the analgesic effect induced by acupuncture in AA model rats. * vs ACU group, P < 0.05.

We used acute adjuvant arthritis (AA) model rats^31^, which were randomly divided into two groups. One group was the normal acupuncture (ACU) group, and the other group was subcutaneously injected with sodium cromolyn (0.02 g/ml, 20 μl, with half receiving 5 mm subcutaneous injections into the muscle and the other half receiving 2 mm subcutaneous injections under the dermis) (CRO + ACU) at local acupoints 5 min before the acupuncture. Sodium cromolyn is a stabilizer of mast cell membranes. Thus, it can block mast cell degranulation that has been induced by acupuncture. Figure 3 shows the tissue sections of the acupoints of the two groups. It is evident that the degranulation phenomenon of the mast cells decreased significantly when acupuncture was administered after the injection of sodium cromolyn at the acupoints, and the statistical data show that such an inhibitory effect was common and reliable, as shown in Fig. 3(d). We measured the pain threshold at the plantar of the ankle joint of the rats in the ACU, Model and CRO + ACU groups using an Analgesia Meter before establishing the model and both before and after the treatment, respectively. Moreover, we normalized the data based on the basal pain threshold before establishing the model, which was used to determine the analgesic effect of acupuncture, and the result is shown in Fig. 4. Model group are used for control of restrain stress. It was shown on the same model rat that the amount and method of injection at ST 36 do not alter pain threshold^27^. The experiment showed that the pain thresholds of the rats in each group decreased dramatically after establishing the model: acupuncture (ACU) treatment significantly elevated the animals’ pain thresholds to produce an analgesic effect, whereas acupuncture after the injection of sodium cromolyn (CRO + ACU) did not have a significant impact on pain thresholds and did not produce analgesic effects.

**Figure 5:**
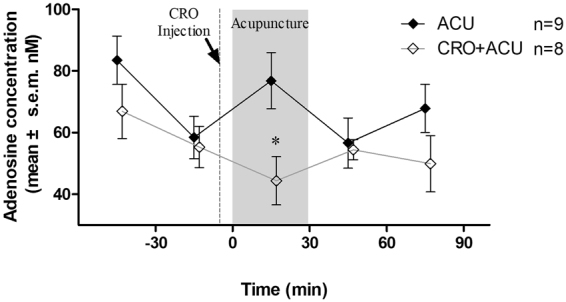
Acupuncture-induced change in adenosine at rat ST36. A microdialysis probe was used to collect tissue fluid specimens at the acupoint, and adenosine concentrations were measured using HPLC. Each data point represents the mean ± s.e.m. of the adenosine concentration in the specimen collected at 30-min intervals. The ACU group was administered 30 min of acupuncture, as represented by the shadow. For the CRO + ACU group, sodium cromolyn solution was injected at the acupoint 5 min before acupuncture, which is represented by the dotted line. Sodium cromolyn was found to inhibit an increase in the adenosine concentration triggered by acupuncture. *vs ACU group, P < 0.05.

To monitor the degranulation of mast cells at the acupoint and the changes in adenosine concentrations at the acupoint, we used the acupoint microdialysis method^23^ to take local samples at rat ST36, and we used high-performance liquid chromatography to measure the adenosine concentrations. The results are shown in Fig. 5. In the experiment, the ACU group received 30 min of acupuncture from 0–30 min, whereas the CRO + ACU group received local subcutaneous injections of sodium cromolyn 5 min before acupuncture. The animals in the two groups had similar local adenosine concentrations at the acupoint before acupuncture (P > 0.05). During acupuncture, the adenosine concentrations in the ACU group increased, which led to significantly higher levels than those observed in the CRO + ACU group (P < 0.05, vs the ACU group), whereas the adenosine concentration of the latter group did not change significantly after acupuncture. The increase of adenosine didn’t last after acupuncture in ACU group, it might relate mainly to the initiation of acupuncture effect. In our previous studies, we found that injection of 20 μl of saline locally at the acupoint using the same methodology did not significantly influence the induction of an acupuncture effect^24^. These results demonstrate that when the activation of mast cells is inhibited, acupuncture can no longer induce an increase in the local adenosine concentration at the acupoint.

**Figure 6:**
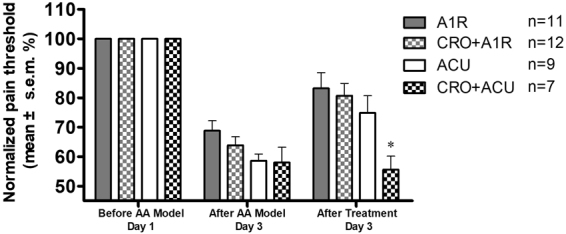
Local injection of sodium cromolyn into ST36 did not inhibit the analgesic effect caused by A1 receptor activation at this acupoint. The pain threshold was normalised according to the pre-modelling pain threshold; the data are presented as the mean ± s.e.m. On day 1, the AA model was established; however, the pre-modelling pain threshold was measured before establishing the model. On day 3, the post-modelling pain threshold was measured first, and the post-treatment pain threshold was measured 20 min after the treatment. For the ACU group, acupuncture was performed at ST36 for 20 min. For the A1R group, CCPA solution was injected locally at the acupoint. For the CRO + A1R group, sodium cromolyn was injected locally at the acupoint 5 min before the injection of CCPA. *vs ACU group, P < 0.05.

The studies by Goldman *et al*. noted that adenosine plays its role at local acupoints through its A1 receptor^23^. To determine whether sodium cromolyn merely inhibits increases in adenosine concentrations rather than inhibits the activation process of acupuncture-related local adenosine A1 receptors, we injected adenosine A1 receptor agonist CCPA into the ST36 acupoint of the AA rat model (A1R group) and compared the results of the action of CCPA when the degranulation of mast cells was blocked (CRO + A1R group). The results are shown in Fig. 6. The injection of the adenosine A1 receptor agonist CCPA alone can have an analgesic effect similar to that of acupuncture, and this effect cannot be inhibited by blocking the degranulation of mast cells through the injection of sodium cromolyn. This suggests that the inhibition of the acupuncture effect by sodium cromolyn occurs by its inhibition of the increase in adenosine concentration, which is caused by the activation of mast cells. Increases in the adenosine concentrations during acupuncture analgesia are regulated by mast cell activation at the acupoints.

### The role of local histamine receptors at the acupoint in acupuncture analgesia

**Figure 7:**
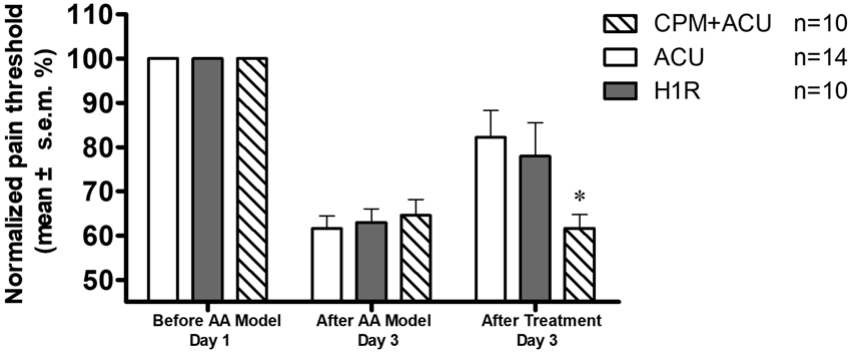
Effects of histamine H1 agonism and antagonism on the acupuncture analgesia. The pain threshold was normalised according to the pre-modelling pain threshold. The data are presented as the mean ± s.e.m. On day 1, the AA model was established; however, before establishing the model, the pre-modelling pain threshold was measured. On day 3, the post-modelling pain threshold was measured first, and the post treatment pain threshold was measured 20 min after treatment. For the ACU group, acupuncture was performed at ST36 for 20 min. For the H1R group, the H1 agonist solution was locally injected at the acupoint. For the CPM + ACU group, the H1 receptor antagonist was locally injected at the acupoint 5 min before acupuncture. Both acupuncture and the activation of the H1 receptor at the ST36 acupoint were found to lead to analgesic effects. The H1 receptor antagonist was found to inhibit the analgesic effect triggered by acupuncture. * vs ACU group, P < 0.05.

During the degranulation of mast cells at an acupoint, a large amount of histamine is released into the tissue with the granules. In our previous studies, we found that a local injection of histamine at the acupoint could lead to an analgesic effect^27^; notably, the histamine H1 receptor is the interaction target of histamine in the peripheral tissues for multiple responses^26^. To determine whether the histamine H1 receptor is involved in the acupuncture analgesic effect, we used specific antagonists and inhibitors of the histamine H1 receptor for further study. Similarly, we used an AA model and used specific agonists and antagonists of the histamine H1 receptor to examine the role of histamine in the acupuncture analgesic effect. As shown in Fig. 7, the ACU group is the acupuncture group. The H1R group was locally injected with the histamine H1 receptor agonist 2-pyridineethanamine dihydrochloride^32^ at the acupoint, which produced an effect similar to the acupuncture analgesic effect. The CPM + ACU group was locally injected with the histamine H1 receptor antagonist chlorprophenpyridamine maleate (CPM)^33^ at the acupoint 5 min before acupuncture. The animals in the CPM group had a lower analgesic effect after acupuncture than did the animals in the H1 receptor agonist group, and there was a significant difference when compared with the acupuncture group (P < 0.05, vs ACU, see Fig. 7). In addition, injection of the H1 receptor agonist alone at the acupoint achieved a similar effect as acupuncture analgesia (P < 0.05), and this had an effect similar to the aforementioned injection of the A1 agonist alone at the acupoint. Thus, although acupuncture can cause the degranulation of mast cells and increases in histamine and adenosine at the acupoint, if a histamine H1 receptor antagonist is injected into the acupoint before acupuncture, the acupuncture analgesic effect will be significantly inhibited. This suggests that the activation of the histamine H1 receptor at the acupoint is a key step in the generation of the acupuncture analgesic effect following local mast cell degranulation, histamine release into tissue and adenosine concentration increases, all three of which are triggered by acupuncture. If the H1 receptor is blocked, then the stimulation signal at the acupoint cannot generate the acupuncture analgesic effect.

### Effects of A1 and H1 receptor activation at the acupoint on β-endorphin in cerebrospinal fluid

**Figure 8:**
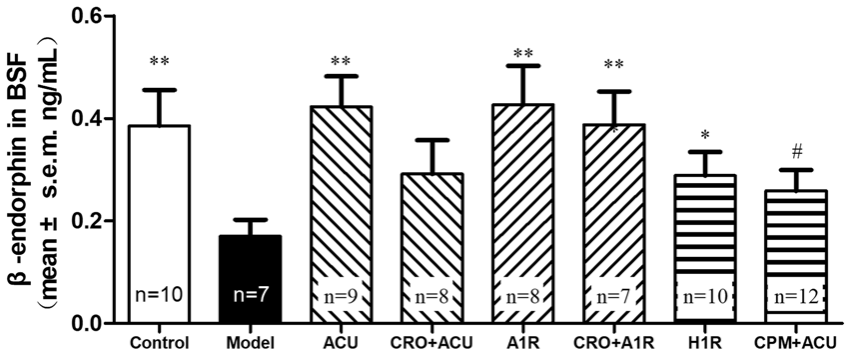
Effects of acupuncture and the influences of mast cells, the A1 receptor and the H1 receptor on β-endorphin in the cerebrospinal fluid of animals. ELISA analysis was used to measure the concentrations of β-endorphin in the cerebrospinal fluid of rats. The Control and Model groups were the blank control and the AA model control, respectively. For the ACU group, acupuncture was performed at ST36 for 20 min. For the H1R group, an H1 agonist solution was locally injected at the acupoint. For the CPM + ACU group, the H1 receptor antagonist was locally injected at the acupoint 5 min before acupuncture. For the A1R group, CCPA solution was injected locally at the acupoint. For the CRO + A1R group, sodium cromolyn was injected locally at the acupoint 5 min before the injection of CCPA. For the CRO + ACU group, sodium cromolyn solution was injected at the acupoint 5 min before the acupuncture. For the CRO + A1R group, sodium cromolyn solution was injected at the acupoint 5 min before the injection of the CCPA solution. The EDP concentration in the model group was found to be significantly lower than that of the blank control group. Acupuncture was shown to elevate the EDP concentration, whereas sodium cromolyn or the H1 receptor antagonist was shown to inhibit such an effect. Direct activation of the H1 receptor was shown to increase the EDP concentration. Activation of the A1 receptor was shown to increase the EDP concentration, whereas sodium cromolyn did not demonstrate the ability to inhibit such an effect. *vs Model P < 0.05; **vs Model P < 0.01; # vs ACU P < 0.05.

The acupuncture analgesic effect relies on the release of multiple endorphins^28^. Because the A1 and H1 receptors of an acupoint play an important role in transmitting the acupuncture analgesic signal, can the activation and blocking of A1 and H1 cause changes in the release of endorphins in the brain? We chose β-endorphin in cerebrospinal fluid as an indicator of the release of endorphins. We used an AA model and established 8 groups, including a blank control group (Control), a model group (Model), an acupuncture group (ACU), an acupuncture-after-blocking-mast-cell-degranulation group (CRO + ACU), an activation-of-the-A1-receptor-after-blocking-mast-cell-degranulation group (CRO + A1R), an A1-receptor-activation group (A1R), an H1-receptor-activation group (H1R) and an acupuncture-after-blocking-H1-receptor group (CPM + ACU). We examined the changes in the EDP concentration in rat cerebrospinal fluid during the treatment, and the results are shown in Fig. 8. As seen in the figure, the animals in the blank control group had a higher EDP concentration in the cerebrospinal fluid, which decreased dramatically after establishing the model. In clinical studies of various types of pain, decreases of EDP concentrations upon the occurrence of pain have previously been established^34^. Acupuncture can cause a significant increase in EDP, reaching or even surpassing the level of a blank control group, which is represented as an analgesic effect in animal behaviour. If mast cell degranulation at the acupoint was inhibited (CRO + ACU) or the H1 receptor at the acupoint was blocked before acupuncture, EDP in the cerebrospinal fluid exhibited slight increases, but no significant differences emerged compared with the model group (P > 0.05, vs Model, see Fig. 8). If the A1 receptor agonist (A1R) is injected at the acupoint, then the same effect as acupuncture can be achieved, and this effect will not be inhibited by blocking mast cell degranulation at the acupoint (CRO + A1R). The experimental result of the changes in these endorphins in the brain is fully consistent with the aforementioned analgesic effect observed in animals. This suggests that the activation of mast cells and the H1 receptor at an acupoint generates an analgesic effect through the central mechanism that unites the peripheral acupoints with the central mechanism.

## Discussion

Mast cells are widely distributed across the skin on the surface of the body and in muscle tissue, and they are a major component of innate immunity. Its involvement in acupuncture had been proved in former studies. During acupuncture, mast cells are activated after deformation of collagen fiber in the acupoint^21^. Its activation effects acupuncture induced peripheral afferent signals^35^ as well as analgesia effects^24^. However in addition to their immunological activation, mast cells can be activated by many other stimulus methods^25^. Studies in recent years have found that many physical stimulation methods, including mechanical stimulation^36^, shearing force^37^, and osmotic pressure^38^, can all lead to mast cell degranulation. Our previous studies have already shown that mast cell activation caused by mechanical stimulation was associated with the TRPV2 channel protein on the membrane of mast cells^22^. The TRPV2 channel protein can be activated by heat and mechanical stimulation; however, the animal pain models constructed using TRPV2 gene knockout mice did not exhibit significant differences relative to wild-type mice^39^. In the present work, we show that after TRPV2 knockout, acupuncture no longer activated local mast cells in the acupoints and acupuncture analgesia was significantly attenuated. Taken together, mechanical stimulation with acupuncture may act directly on the TRPV2 channel on the mast cells and lead to mast cell degranulation, thereby triggering an analgesic effect. The activation method of mast cells determines the mechanism by which they contribute to an acupuncture effect. We found that during acupuncture, mast cells could be directly activated by stimulation via acupuncture and that mast cells play the role of a transducer in converting acupuncture physical stimulation to biochemical signals. The relation between mast cells and acupuncture effects may be beyond this specific model since mast cells are activated by acupuncture in other disease models^40,41^.

The adenosine concentration in tissue is determined by the metabolic balance of adenosine substances^42^. ATP in tissue is catabolized into adenosine through the catalysis of CD39 and CD37 on the cell membrane. The equilibrative nucleoside transporters on the nearby cell membrane carry out bidirectional transport to regulate the adenosine concentration in the tissue; therefore, the rapid release of both ATP and adenosine can elevate the local adenosine concentration. The activation of mast cells elevates the local adenosine concentration through direct and indirect methods. *In vitro* experiments have shown that various mast cells release adenosine after their activation^43^, wherein mast cells have been activated through their A3 receptors^44^ to form a positive feedback effect to further induce the direct release of adenosine. In addition to this direct release, activated mast cells can elevate ATP release from endothelial cells in tissue by releasing lipopolysaccharide^45^ and, thus, indirectly promote the increase of adenosine concentrations. Goldman *et al*. found that during acupuncture, the local adenosine concentration at an acupoint is increased, and A1 receptor mediates the acupuncture effects on mice^23^. However, they could not provide an explanation for the source of such adenosine and its mechanism of expression. Mast cells are unevenly distributed in human body surface tissue^46^, and they are especially prevalent at acupoints^47^. We found an increase in the local adenosine concentration during acupuncture. After the activation of mast cells was inhibited, acupuncture could not induce such an increase in adenosine concentration. We also noted the association between adenosine signalling stimulated by acupuncture and mast cells. Mast cells are transducers for acupuncture at acupoints, from which the release of adenosine is, at minimum, an important factor in establishing an initiation signal at the acupoints during acupuncture analgesia.

Cellular approach showed that mast cells release ATP in response to multiple types of physical stimulations^48^. The current study provides *in vivo* evidence for its release during acupuncture. However, as indicated by Tang *et al*.^49^, P2X receptors, which are activated by ATP, might be modulated only as a consequence for anti-nociception. Adenosine, through A1 receptors, plays a more important role in the acute analgesia effects. Our results showed that it is modulated by mast cells in the acupoint.

Histamine in tissue derives mainly from basophils and mast cells, and its major function in body surface tissue is reflected in the immune response. Histamine is stored in granules in mast cells. Degranulation of mast cell release histamine to extracellular space. Histamine increases vascular permeability through the histamine H1 receptor to activate nerve endings, relax vascular smooth muscle, and cause redness and itching^26^. Davis *et al*. also showed that activation of the histamine H1 receptor can stimulate cutaneous afferent excitability and cause itching^50^. On the other hand, gene knockout experiments further support the view that the H1 receptor is involved in the pain sensation process^51^, but its targeting receptor and mechanism were still unclear. In this study, we found that injecting an H1 receptor agonist alone could induce the analgesic effect and that an H1 receptor antagonist could inhibit the acupuncture analgesic effect. This suggests that during acupuncture, the histamine released from the activated local mast cells at acupoints plays a role in the acupuncture effect through the H1 receptor. Therefore, histamine and the H1 receptor may compose an important biological signalling pathway during acupuncture analgesia.

The release of enkephalin, endorphin, dynorphin and many endogenous opioid peptides is the central pathway to achieve the acupuncture analgesic effect^52,53^; however, different acupoints and stimulation methods may yield differential effects in the release of endogenous opioid peptides^28^. Studies have found that acupuncture can cause an increase in the β-endorphin concentrations in the cerebrospinal fluid^29^ and that an increase of the β-endorphin concentration in the central nervous system can lead to an increase in the pain threshold^54^. However, no report has been made on the relationship between the peripheral local changes caused by acupuncture and the central release of opioid peptides. Here, we chose β-endorphin as an indicator of a central response to the acupuncture analgesic effect. We found that local peripheral H1 and A1 receptor agonists produced effects similar to that of acupuncture in terms of leading to an increase of the β-endorphin concentrations in cerebrospinal fluid, whereas blocking mast cell degranulation at acupoints or injecting an H1 receptor antagonist could inhibit the increase of β-endorphin caused by acupuncture. Such a change in the β-endorphin concentration was consistent with the acupuncture analgesic effect. This result further suggests that the activation of local peripheral adenosine and histamine receptors has a central analgesic mechanism similar to that of acupuncture.

The above results and former studies suggest a pathway of acupuncture analgesia, in which physical mechanical stimulation generates an overall biological effect in the body. Needle penetration with the action of manipulation leads to the winding and deformation of collagen fibres in the connective tissue^18,21^. Acupoint tissue is often enriched with mast cells^24^, and under the action of high stress, TRPV2 proteins open to induce mast cell activation (mechanical activation) and degranulation^22^. The major component of the granules, histamine, is released into the tissue to cause increases in histamine and adenosine concentrations in local tissue^23^. Adenosine binds to the A3 receptors on the membranes of other nearby mast cells to generate positive feedback, thereby inducing continued mast cell degranulation (chemical activation) and the release of more histamine and adenosine. Histamine and adenosine will bind to the H1 and A1 receptors on the nerve receptor, respectively, to generate specific excitatory nerve signals^35,55,56^, and such a neural electrical signal is transmitted to the central nervous system for integration, which results in the release of opioid peptides, including β-endorphin, in the brain, thereby generating an long lasting analgesic effect after the removal of needle^16^.

This paper focuses on the roles of mast cells and key bioactive substances in the initiation of acupoints. We used gene knockout, specific receptor antagonists, agonists, and other technical means to study the acupuncture analgesic effect triggered by mast cell activation and adenosine concentration changes at acupoints and to study the changes in β-endorphin in cerebrospinal fluid. We found that the mechanosensitive protein TRPV2 was involved in local mast cell activation at the acupoint caused by acupuncture and the generation of the analgesic effect. Acupuncture can influence increases in adenosine concentrations in local tissue by activating mast cells, and the histamine that is released due to the activation of mast cells plays a role in the acupuncture effect via the histamine H1 receptor. Moreover, the local activation of the A1 and H1 receptors at acupoints will lead to an increase of β-endorphin in cerebrospinal fluid. Through the study reported in this paper, we proposed a signal initiation pathway involving collagen propose based on an acupoint-mast cell-TRPV protein axis, clarified the targets of the important initiation substances (namely, histamine and adenosine) at the acupoints, and elucidated the cellular and molecular biological mechanisms of the acupoint initiation on the acupuncture effect. These findings will form a new frontier in the cellular and molecular biological studies of the acupuncture analgesic effect.

## Methods

### Acute adjuvant arthritis model rats

Clean SD male rats were provided by the Shanghai Experimental Animal Breeding Center of the Chinese Academy of Sciences, under license number SCXK (Hu) 2007-0005. The body weight of the rats was approximately 150 ± 20 g, and all rats were in good health. All rats were randomly grouped and numbered, and they were fed and housed under standard conditions (GB14925-2001). All animal experimental methods were approved by the Experimental Animal Ethics Committee of the Shanghai Acupuncture and Meridian Research Center. All methods were performed in accordance with the committee’s guideline and regulation.

The basal pain threshold values (before AA model) of all animals were measured on the day following their transport to the lab, which for all the groups—except the blank control group—was one day prior to that upon which they were modelled. The method for establishing the models was to use 10% chloral hydrate for intraperitoneal injection according to 0.04 ml/100 g to anaesthetise the animals, after which 0.05 ml of CFA was injected into the left ankle joint cavity. On the second day of modelling (the fourth day after arrival), by direct observation, the rats that were determined to have significant swelling at the modelling site and to have problems moving around were considered to have undergone successful modelling. The post-modelling pain threshold (after AA model) was measured immediately, and the blank control group was also measured during the same time frame. After measuring the post-modelling pain threshold, each group was treated differently. Treated rats were placed in an Analgesia Meter, and 20 min later, the post-treatment pain threshold value (after treatment) measurement was started.

### TRPV2 gene knockout model

The TRPV2 gene knockout male mice used in this study were brought from the Shanghai Research Center for Model Organisms. The gene knockout site was the partial sequence of the fourth exon of the TRPV2 gene, and a conditional knockout vector plasmid was constructed. Gene targeting was performed in SCR012 embryonic stem cells, with screening performed for the embryonic stem cell clones to confirm that both arms had the correct homologous recombination. The positive clones were injected into the embryo sac of normal C57BL/6 J mice, which developed into chimeric parents and bred with C57BL/6 J to obtain mice with both arms being positive. Eventually, such heterozygous mice were crossed with E2A-Cre mice to obtain systemic gene knockout mice.

Basal pain threshold was measured on the first day for all animals (before pain model). After the measurement, the animals in all groups underwent modelling treatment. The modelling method was to use 0.3% pentobarbital sodium for intraperitoneal injection according to 1.2 ml/100 g to anaesthetise the animals, after which 0.03 ml of CFA (Complete Freunds’s Adjuvant, SIGMA, USA) was injected into the left hind feet to execute the inflammatory pain model. On the second day of modelling, by direct observation, the mice that were determined to have significant swelling at the modelling site and problems moving around were considered to have undergone successful modelling. The post-modelling pain threshold value (after pain model) was measured immediately. After the mice were treated with acupuncture for 20 min, the pain threshold value (after acupuncture) after acupuncture was measured.

### Determination of the animal pain threshold

An Analgesia Meter (IITC INC. Life Science Instrument, USA) was used to stimulate the plantar near the left ankle joint of a rat, and its hindpaw withdrawal latency was measured. For mice, the bottoms of their left feet were stimulated, and their hindpaw withdrawal latencies were measured. Before the measurement started, all animals were left for 20 min in a transparent box (with freedom of movement), which would subsequently be used in the experiment, to permit adaptation to the environment. The ambient temperature was maintained at 24 ± 2 °C, the interval between measurements was 10 min, and the measurement was performed three times, from which an average was calculated. To protect the animals from burns, the upper limit of the instrument was set at 20 s.

### Acupuncture stimulation

The acupuncture position was the Zusanli (ST36) on the left leg, and the acupuncture method consisted of 30 s of lifting-thrusting, needle retention for 30 s, 30 s of twisting, and needle retention for 30 s, which were performed alternately. After the acupuncture had been complete for 20 min, the post-treatment pain threshold value (after AA model) was measured. The AA model rats received 20 min of acupuncture treatment in the pain-threshold and EDP-measurement experiments versus 30 min of acupuncture treatment in the microdialysis experiment. In the mouse experiment, the acupuncture time was 30 min.

### Operation in each group

For the pain threshold test and the EDP test, the rats were randomly divided into the following groups: the Control, which received no treatment; the Model group, in which AA model rats were restrained for 20 min; the ACU group, which received acupuncture at ST36 for 20 min; the H1R group, which was injected with 2-pyridineethanamine dihydrochloride (200 μg/ml, 50 μl; H1 agonist) at ST36; the CPM + ACU group, which was injected with chlorprophenpyridamine maleate (50 μl, 0.4 mg/ml; H1 antagonist) at ST36, 5 min before acupuncture; the A1R group, which was injected with CCPA (0.04 mg/ml, 20 μl) at ST36; and the CRO + ACU and CRO + A1R groups, which were injected with sodium cromolyn (0.02 g/ml, 20 μl) at ST36, 5 min before acupuncture or CCPA injection.

### Tissue sectioning and mast cell staining

After measuring its AT value, the rat was immediately sacrificed by cervical dislocation. With the acupoint as the centre, a 5 × 5 × 5 mm specimen of skin and muscle tissue was taken, which was fixed in formalin solution for 48 hours. The tissue was dehydrated using a tissue dehydration machine, and paraffin was used to embed the tissue. The tissue was sectioned at a thickness of 5 μm. Toluidine blue (TB) was used to stain the mast cells.

Under a 400× upright dissecting microscope, the total number of mast cells and the number of degranulated mast cells were counted. Mast cells with a blurred boundary, blue staining of the surrounding region, and significant degranulation were counted as degranulated mast cells. The ratio of the number of degranulated mast cells versus the total number of mast cells in one section was used as the degranulation rate. For each animal, three random selected sections were counted, and the average was taken.

### Microdialysis and the HPLC analysis of adenosine

To achieve stable anaesthesia during the 3 hours experiment, isoflurane is used as anaesthetic. The rats were anaesthetised by connecting them to an isoflurane evaporator (Jiangsu Taixing AoKai Medical Instrument Co., Ltd) through an animal ventilator (Harvard, USA). After induction, the animals were continuously anaesthetised using an inhalation of 1.5% isoflurane, and 30 min later, the inhalation was reduced to 1.0% isoflurane, which was maintained until the experiment was completed. A microdialysis probe (MD2211, Basi, USA) was planted subcutaneously at the acupoint, and the dialysis solution used was Ringer’s solution. A syringe pump was used to provide power, the flow rate was set at 2 μl/min, and one sample was taken every 30 min, lasting for 2.5 h, to yield a total of 5 samples.

High-performance liquid chromatography was performed using an Agilent 1100 system. The mobile phase was a methanol-water solvent pair, of which the water phase contained 50 mmol/L monosodium phosphate and 4 mmol/L disodium phosphate, with a ratio between methanol and water of 5:95. The flow rate was 0.8 ml/min; the chromatography column was an Agilent odx with an inner diameter of 4.6 mm, a column length of 250 mm, and a filling gap of 5 μm; and the detector was a UV detector with a detection wavelength of 256 nm. The adenosine standard was checked both before and after the measurement, and the concentrations were 3000 nM, 1000 nM, 300 nM, and 100 nM. The detection results of the animal samples were compared to the standard according the peak areas, and the concentration was calculated.

### Detection of β-endorphin by ELISA

Larger male rats at 200 ± 20 g were used to facilitate collection of CSF. Injections and acupuncture were performed on the rats while restrained in a Broome style restrainer. After treatment, the animals were anaesthetised by intraperitoneal administration of chloral hydrate. The foramen magnum was exposed, and a 1-ml syringe with a bent tip was used for intradural collection of CSF. All CSF was then stored in a −80 °C refrigerator and was diluted 3 times before use in the ELISA test. An ELISA kit (S-1170, Peninsula Laboratories International, Inc., USA) was used for the detection qualification of β-endorphin, with the test performed according to the manufacturer’s manual.

### Methods for data analysis

The mast cell degranulation rate met homogeneity of variance assumptions between the groups, and a Independent-sample two-tail T-test was used for the analysis with P < 0.05 considered a statistically significant difference. The rat pain threshold data were normalised to each animal’s basal pain threshold value. Single-factor analysis of variance was used for the comparison between the groups, with P < 0.05 regarded as indicative of a significant difference. The mice were measured three times for their pain threshold, and the average of these measurements was calculated. Independent-sample two-tail T-tests were used for comparisons between the groups, with P < 0.05 considered indicative of a significant difference. Independent-sample two-tail T-tests were used to compare the local adenosine concentration changes at the acupoint between groups, with P < 0.05 considered indicative of a significant difference. Independent-sample single-tail T-tests were used to compare the endorphin concentrations in the cerebrospinal fluid between groups, with P < 0.05 considered indicative of a significant difference.
